# Lamin A/C Mechanosensor Drives Tumor Cell Aggressiveness and Adhesion on Substrates With Tissue-Specific Elasticity

**DOI:** 10.3389/fcell.2021.712377

**Published:** 2021-09-14

**Authors:** Enrica Urciuoli, Valentina D’Oria, Stefania Petrini, Barbara Peruzzi

**Affiliations:** ^1^Multifactorial Disease and Complex Phenotype Research Area, IRCCS Bambino Gesù Children’s Hospital, Rome, Italy; ^2^Confocal Microscopy Core Facility, Research Center, IRCCS Bambino Gesù Children’s Hospital, Rome, Italy

**Keywords:** Lamin A/C, mechanosensors, organ tropism, cell spreading, adhesion, tissue stiffness, PDMS substrates

## Abstract

Besides its structural properties in the nucleoskeleton, Lamin A/C is a mechanosensor protein involved in perceiving the elasticity of the extracellular matrix. In this study we provide evidence about Lamin A/C-mediated regulation of osteosarcoma cell adhesion and spreading on substrates with tissue-specific elasticities. Our working hypothesis is based on the observation that low-aggressive and bone-resident SaOS-2 osteosarcoma cells express high level of Lamin A/C in comparison to highly metastatic, preferentially to the lung, osteosarcoma 143B cells, thereby suggesting a role for Lamin A/C in tumor cell tropism. Specifically, LMNA gene over-expression in 143B cells induced a reduction in tumor cell aggressiveness in comparison to parental cells, with decreased proliferation rate and reduced migration capability. Furthermore, LMNA reintegration into 143B cells changed the adhesion properties of tumor cells, from a preferential tropism toward the 1.5 kPa PDMS substrate (resembling normal lung parenchyma) to the 28 kPa (resembling pre-mineralized bone osteoid matrix). Our study suggests that Lamin A/C expression could be involved in the organ tropism of tumor cells, thereby providing a rationale for further studies focused on the definition of cancer mechanism of metastatization.

## Introduction

Lamins are nuclear type V intermediate filament protein that bridge chromatin to the inner nuclear membrane by forming a protein scaffold at the nuclear periphery, called the nuclear lamina (Gruenbaum et al., 2005; Dahl and Kalinowski, 2011; Gruenbaum and Foisner, 2015). Lamins are highly conserved in all multicellular organisms and in mammals they are classified into two groups: A-type lamins, encoded by *LMNA* gene and generated by alternative splicing into Lamin A, Lamin C and less abundant isoforms, and B-type lamins, which are encoded by the *LMNB1* and *LMNB2* genes that generate Lamin B1 and Lamin B2/B3 isoforms, respectively (Ho and Lammerding, 2012). A- and B-type lamins differ greatly in their expression patterns, biochemical features, and behavior during mitosis. The A-type lamins are among the most mutated human genes, and their mutations cause genetic disorders termed laminopathies, affecting muscle, fat, neuron, bone, and skin tissues, and ranging from muscular dystrophies to lipodystrophies neuropathies and early aging diseases (Worman and Bonne, 2007).

Besides their structural roles in providing a mechanical support to the nucleoskeleton, lamins have been recently recognized as regulators of gene expression and mechanical sensors for tissue elasticity. Several works have clearly demonstrated that lamin A expression functions as a “mechanostat,” revealing that matrix stiffness and mechanical stress are able to regulate Lamin A expression, thereby stabilizing the nucleus and contributing to cell lineage determination (Swift et al., 2013; Swift and Discher, 2014; Ivanovska et al., 2017; Xia et al., 2018; Cho et al., 2019; Gumina et al., 2021).

A- and B-type lamin altered expression have been recently explored in cancer development, progression and propagation, as they are involved in several mechanisms at the basis of tumorigenesis, as epigenetic regulation of gene expression (Hutchison, 2002; Hutchison and Worman, 2004; Melcer et al., 2012), chromosomal numeric stability and aneuploidy (Capo-Chichi et al., 2011). Several reports have suggested their involvement in prostate cancer, hepatocarcinoma, breast and lung cancers (Lim et al., 2002; Kong et al., 2012; Wazir et al., 2013; Irianto et al., 2016; Kaspi et al., 2017). It is worth to note that although Lamin A/C deregulation has been related to cancer onset and progression (Irianto et al., 2016; Dubik and Mai, 2020), conflicting results have been achieved in dependance on the specific tumor assessed (Foster et al., 2010), making difficult to define the nature of lamin function in cancer as a favoring or counteracting factor. Indeed, lamin expression can be variable between and even within cancer subtypes, as we have recently demonstrate in osteosarcoma: our findings demonstrated that lamin expression in human osteosarcoma cells scales with tumor aggressiveness in an integrated mechanism involving MKL1 nuclear shuttling and actin polymerization, as well as pRb expression and YAP nuclear content (Urciuoli et al., 2020). Interestingly, although cancer development in laminopathic patients is a rare event, osteosarcoma is the only neoplasm associated to cases of HGPS and WS syndromes (King et al., 1978; Okamoto et al., 1997; Ishikawa et al., 2000; Shalev et al., 2007).

Osteosarcoma, the most common primary malignant bone tumor in children and adolescents (Sweetnam, 1982; Damron et al., 2007), is a highly aggressive cancer that metastasizes primarily to the lung (Dunn and Dehner, 1977; Kansara and Thomas, 2007). In the present research report, we demonstrated that high metastatic osteosarcoma cells, expressing basal low levels of Lamin A/C, are preferentially spread on Polydimethylsiloxane (PDMS) support resembling normal lung parenchyma, and that Lamin A/C reintegration induces a preferential interaction with the bone matrix-resembling PDMS support and reduces aggressiveness.

## Methods

### Cell Cultures

Pediatric human osteosarcoma SaOS-2 (HTL01001) and 143B (CRL-8303) cell lines were purchased, respectively from Banca Biologica and Cell Factory (IRCCS Azienda Ospedaliera Universitaria San Martino-IST, Genova, Italy) and American Type Culture Collection (Manassas, VA) and grown in Dulbecco’s modified Eagle’s medium (DMEM) (Euroclone) supplemented with 10% Fetal Bovine Serum (FBS), 100 units/ml penicillin/streptomycin (Euroclone) and maintained at 37°C in 5% CO2. The two cell lines have very different aggressiveness, metastatic behavior and organ tropism (Mohseny et al., 2011).

### Lamin A/C Over-Expression

For lamin A/C over-expression in 143B cells, the pBABE-puro-GFP-wt-lamin A was a gift from Tom Misteli (Addgene plasmid #17662^1^; RRID:Addgene_17662) (Scaffidi and Misteli, 2008), and the empty vector pBabe-Puro-GFP was used as control (mock transfection). An amount of 5 × 10^5^ 143B cells was plated in a 10 cm^2^ Petri dish and transfected with 3 μg of plasmid DNA by Lipofectamin 2,000 (Invitrogen), according to the manufacturer’s instructions. Transfected cells were selected by puromycin treatment (5 μg/ml) for 2 weeks and then assessed.

### Immunofluorescence

5 × 10^3^ cells were seeded into plastic-chambered glass microscope slides (BD Falcon), fixed with 4% paraformaldehyde in PBS for 10 min followed by PBS/Triton 0.1% for 5 min. The following primary antibodies were used according to manufacturer instructions: mouse anti-lamin A/C antibody and goat anti-Integrinβ-3 were from Santa Cruz Biotechnology (Temecula, CA, United States), rabbit anti-lamin B1 was from Abcam (Cambridge, United Kingdom), mouse anti-α-tubulin was from Cell Signaling Technology (Danvers, MA, United States). Secondary antibodies conjugated with Alexa Fluor-555 and -647 dyes (Life technologies) were used diluted in 1% PBS/BSA for 1 h, at RT. F-actin and nuclei were stained using Phalloidin-TRITC and Hoechst 33342 (Life Technologies), respectively. Slides were mounted with PBS/glycerol 1:1. Negative controls were performed in each labeling using 1% PBS/BSA without the primary antibody, to verify specific staining.

### Confocal Microscopy and Image Analysis

Confocal microscopy was performed on a Leica TCS-SP8X laser-scanning confocal microscope (Leica Microsystems, Mannheim, Germany) equipped with tunable white light laser (WLL) source, 405 nm diode laser, 3 Internal Spectral Detector Channels (PMT) and 2 Internal Spectral Detector Channels (HyD) GaAsP. Sequential confocal images were acquired using a HC PL APO CS2 40x oil-immersion objective (1.30 numerical aperture, NA, Leica Microsystems) or 63x oil-immersion objective (1.40 NA) with a 1,024 × 1,024 format, scan speed 400 Hz, z-step size of 0.3 μm, with an electronic zoom magnification up to 2.7. Z-reconstructions are imported into LASX 3D Analysis (Leica Microsystems) software to obtain their three-dimensional surface rendering to a better visualization of cytoskeletal architectures. The quantitative analysis of stained cell samples was performed on single confocal images acquired at the adhesion plane of cells, in which the phalloidin positive filopodia were perfectly visible. The mean fluorescence intensity of LMNA/C, LMNB1, ITGB3 and a-TUB was measured by ImageJ software in single section images (40x magnification) randomly selected and analyzed for each cell sample. Tables of images were processed using Adobe Photoshop CS4 software (Adobe Systems Inc.).

### qRT-PCR

Total RNA was extracted from cultured cells using the standard Trizol procedure. Each RNA sample was quantified by NanoDrop 2000 (Thermo Fisher Scientific, Foster City, CA, United States). Two μg of RNA were reverse transcribed using the SuperScript^TM^ II Reverse Transcriptase (Invitrogen- Thermo Fisher Scientific, Foster City, CA, United States) to generate cDNA. The PCR reaction has been carried out with Power SYBR Green dye chemistry (Applied Biosystems by Thermo Fisher Scientific) using the 7,500 Fast Real-Time PCR System (Applied Biosystems, Foster City, CA). Results have been normalized to human GAPDH levels using the 2-ΔΔCt method. Primer pairs 5′–3′: human GAPDH reverse: AGGGGTCTACATGGCAACTG; human GAPDH forward: CGACCACTTTGTCAAGCTCA; human LMNA reverse: GGTCGAAGGACAGAGACTGC; human LMNA forward: CTACACCAGCCAACCCAGAT; human LMNB1 reverse: GGCATCATGTTGCTCTCTCA; human LMNB1 forward: AACGAGACCAGAAGGAAGCA.

### Proliferation Assays

For proliferation assay, LMNA and mock transfected 143B cells were seeded at a density of 500 cells/well in 96-well plates and maintained for 72 h. Cell proliferation was analyzed using the XTT assay kit (ROCHE, Mannheim, Germany) according to the manufacturer’s instructions. Briefly, 50 μl of the activated XTT solution were added to each well and incubated for 4 h. Next, the absorbance of samples was measured with a spectrophotometer (ELISA reader) at a wavelength of 450 nm and a reference wavelength of 650 nm. For crystal violet staining, LMNA and mock transfected 143B cells were seeded into 96-well plates (1 × 10^4^/well) and incubated at 37°C for 72 h, then cells were washed twice in PBS and fixed in cold 100% methanol at room temperature for 10 min. Cells were air-dried and stained with crystal violet for 10 min. After the cells were washed and air-dried, a solution of 50% methanol and 1% SDS was used to dissolve the crystal violet, and optical density (OD) was measured at 595 nm with a spectrophotometer.

### Wound Healing Assay

LMNA and mock transfected 143B cells were cultured at density of 5 × 10^3^/ml in Culture-Inserts (Ibidi). Once reached the confluence, the insert was removed thereby creating a 500 μm wound between the two cell fronts and the complete medium was changed in starvation medium (DMEM medium supplemented with 0.25% of BSA) to reduce cell proliferation. Following 24 h, cells were fixed with 4% paraformaldehyde, stained with the nuclear dye Hoechst and the wound size was analyzed using ImageJ software.

### Experiments on PDMS Substrates With Tissue-Specific Elasticity

For adhesion and spreading analysis on substrates with tissue-specific elasticity, Polydimethylsiloxane (PDMS)-coated glass cover-slips (IBIDI μ-Dish 35 mm High ESS, 1.5 and 28 kPa) were used. PDMS dishes were coated with a thin layer of 1 mg/ml Collagen I from calf skin (Sigma-Aldrich) for 30 min, then allowed to dry and sterilized under Ultraviolet (UV) light. LMNA- and mock-transfected 143B cells were cultured in PDMS dishes with a stiffness of 1.5 and 28 kPa in complete medium, resembling, respectively, bone osteoid matrix (Engler et al., 2006) and normal lung parenchyma (Liu et al., 2010). Following 24 h, cells were fixed with 4% paraformaldehyde, stained with the F-actin dye Phalloidin-TRITC (Life technologies) and the cell morphology and spreading analyzed using ImageJ and Filoquant softwares.

### Statistical Analysis

Data were referred from at least three independent experiments for each condition. All analyses were completed using GraphPad Prism 6.0. Differences in the level of fluorescence intensity distributions were evaluated with Kruskal-Wallis Mann-Whitney rank-sum test and Dunn’s multiple comparison *post hoc* test. Differences between LMNA/C and mock transfected 143B in terms of proliferation, migration (wound healing assay) and cellular parameters were evaluated by one-way analysis of variance. *P*-values < 0.05 were considered statistically significant.

## Results

### Lamin A/C Is Differentially Expressed in Osteosarcoma Cells With Different Aggressiveness and Tissue-Specific Tropism

**FIGURE 1:**
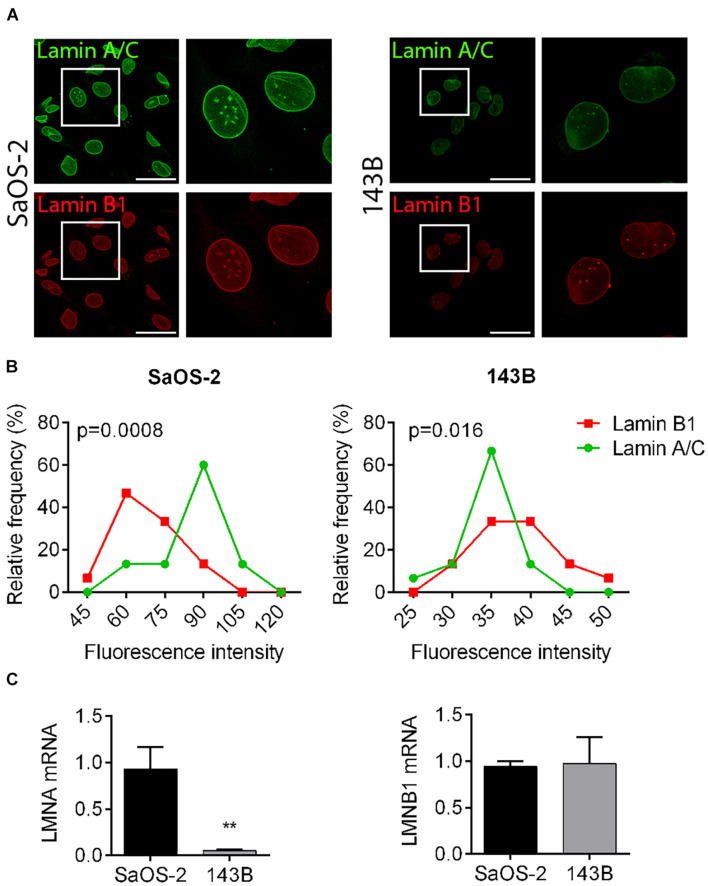
Lamin A/C and B1 expression in osteosarcoma cell lines with different aggressiveness. **(A)** Representative pictures of the immunofluorescence analysis of Lamin A/C (green) and Lamin B1 (red) in SaOS-2 and 143B cells. Bar: 50 μm, *n* = 100. **(B)** Frequency distribution of fluorescence intensities of Lamin A/C (green line) and Lamin B1 (red line) in SaOS-2 and 143B cells. **(C)** mRNA expression of LMNA and LMNB genes in SaOS-2 and 143B cells, normalized versus GAPDH gene expression. **p < 0.01.

Low aggressive and “bone matrix resident” SaOS-2 cells express a high amount of Lamin A/C in comparison to highly metastatic and “lung tropic” 143B cells, as assessed by confocal microscope imaging evaluation and quantification. Also Lamin B1 levels resulted reduced in 143B cells in comparison to SaOS-2 cells, although with a lower extent (Figures 1A,B). Lamin A/C differential amounts in osteosarcoma cells were also confirmed at a transcriptional level by qRT-PCR analysis (Figure 1C), prompting us to further analyze the effects of LMNA gene modulation in tumor cells aggressiveness.

### Lamin A/C Over-Expression in 143B Cells Reduces Tumor Aggressiveness

**FIGURE 2:**
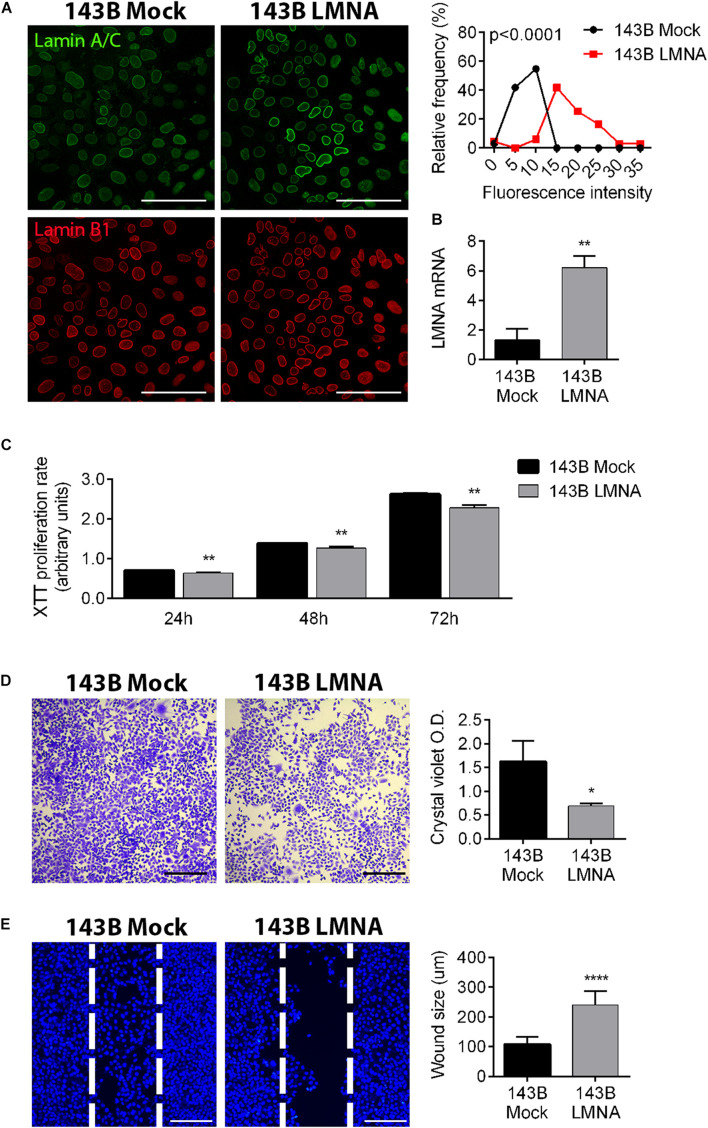
Modulation of Lamin A/C expression in 143B cells alters tumor phenotype. **(A)** Confocalmicroscope imaging of Lamin A/C (pseudo-colored in green) and Lamin B1 (red) in mock-transfected (143B mock) and Lamin A/C-transfected (143B LMNA) 143B cells. Assessment of the relative frequencies of immunofluorescence intensities of Lamin A/C in 143B Mock (black line) and 143B LMNA (red line) cells. Bar: 100 μm, *n* = 100. **(B)** mRNA expression of LMNA gene in mock-transfected (143B mock) and Lamin A/C-transfected (143B LMNA) 143B cells, normalized versus GAPDH gene expression. **p < 0.01. **(C)** XTT proliferation assay performed at 24, 48, and 72 h on mock-transfected (143B mock) and Lamin A/C-transfected (143B LMNA) 143B cells. **p < 0.01. **(D)** Representative pictures of crystal violet staining of 143B Mock and 143B LMNA cells (left panels) and the relative quantification (right panels) as an evaluation of cell proliferation at 72 h after plating. Bar: 500 μm. *p < 0.05. **(E)** Representative pictures of the wound healing assay performed on 143B Mock and 143B LMN-A cells stained with the nuclear dye Hoechst and assessed at 24 h after Ibidi insert removal (left panels). The white dashed line represents the position of cell migrating front at Ibidi insert removal (*T* = 0). Bar: 200 μm. ****p < 0.001.

To demonstrate that the reintegration of Lamin A/C expression in 143B cells is sufficient to attenuate tumoral aggressiveness and restore a “bone matrix tropism” phenotype, we over-expressed *LMNA* gene in these cells by plasmid transfection (LMNA transfected cells), using a mock transfection as control (mock transfected cells). Figure 2A demonstrates a significant higher expression of Lamin A/C in transfected cells in comparison to mock-transfected cells, also confirmed at a transcriptional level by RT-PCR (Figure 2B). LMNA transfected 143B cells show a significant reduction of proliferation in comparison to mock-transfected cells as evaluated in a time-course experiments at 24, 48, and 72 h by XTT proliferation assay (Figure 2C) and confirmed by Crystal violet staining as an assessment of cell count at 72 h (Figure 2D). Moreover, LMNA transfected 143B cells result less aggressive in terms of migration capability, showing a statistically larger wound size than that achieved by mock-transfected 143B cells in a 24-h wound healing assay (Figure 2E). All together, these results demonstrate a reduction of tumor-associated features in 143B cells following Lamin A/C restoration.

### Reintegration of Lamin A/C in 143B Cells Reverts Cell Adhesion and Spreading on Substrates With Tissue-Specific Elasticity

**FIGURE 3:**
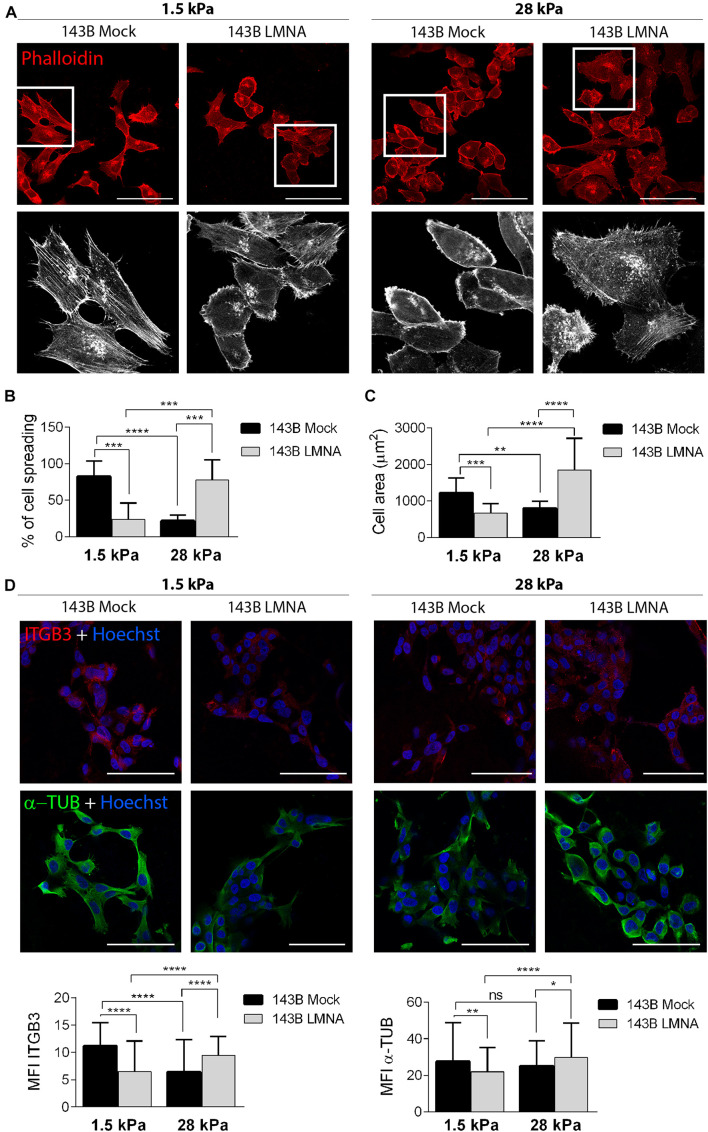
Adhesion of 143B cells on PDMS substrates with different elasticity. **(A)** Confocal microscope representative pictures of 143B Mock and 143B LMNA cells stained with phalloidin (red) and cultured on PDMS substrates with a 1.5 and 28 kPa elasticity (upper panels). Bar: 100 μm. *n* = 50. High magnification of the boxed area showing phalloidin staining pseudo-colored in white to improve cell spreading details (lower panels). **(B)** Quantification of the percentage of cell spreading in 143B Mock and 143B LMNA cells cultured on the 2 different PDMS substrates. **(C)** Measurement of the cell area of 143B Mock and 143B LMN-A cells cultured on the 2 different PDMS substrates. **p < 0.01; ***p < 0.005; ****p < 0.001. **(D)** Confocal microscope representative pictures of 143B Mock and 143B LMNA cells immunostained for ITGB3 (red), α-TUB (green) and counterstained with nuclear dye Hoechst (blue) and cultured on PDMS substrates with a 1.5 and 28 kPa elasticity (upper panels). Bar: 100 μm. *n* = 50. Quantification of the mean fluorescence intensity (MFI) of ITGB3 and α-TUB in 143B Mock and 143B LMNA cells cultured on the 2 different PDMS substrates. ns, not significant; *p < 0.05; **p < 0.01; ****p < 0.001.

**FIGURE 4:**
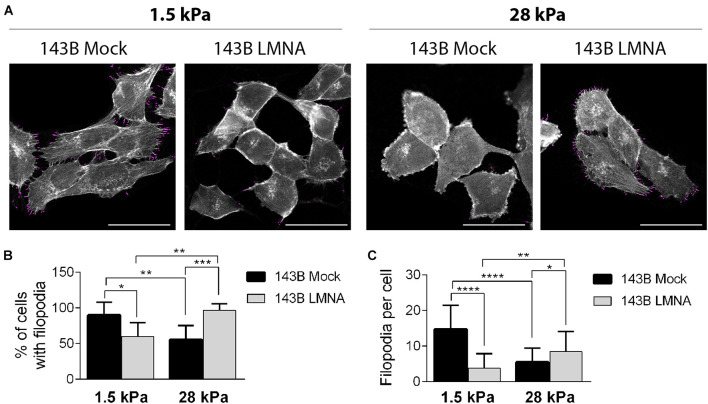
Evaluation of 143B cell adhesion on the two different PDMS substrates. **(A)** Representative pictures obtained by ImageJ Filoquant analysis performed on 143B Mock and 143B LMNA cells stained with phalloidin (pseudo-colored in white) and cultured on PDMS substrates with a 1.5 and 28 kPa elasticity Filopodia resulted by Filoquant analysis are shown in purple staining. Bar: 40 μm, *n* = 50. Relative quantification of% of cell with filopodia **(B)** and filopodia density **(C)** derived from ImageJ Filoquant analysis performed on 143B Mock and 143B LMN-A cells cultured on PDMS substrates with a 1.5 and 28 kPa elasticity. *p < 0.05; **p < 0.01; ***p < 0.005; ****p < 0.001.

PDMS dishes with a stiffness of 1.5 and 28 kPa were used to mimic lung parenchyma and bone matrix, respectively. LMNA- and mock-transfected 143B cells were cultured on 1.5 and 28 kPa PDMS dishes and assessed for adhesion and spreading. Phalloidin staining demonstrates an opposite behavior between mock- and LMNA-transfected 143B cells on the two different PDMS substrates, the former being more adherent and spread to 1.5 kPa substrate, and the latter to the 28 kPa substrate, as demonstrated by confocal microscope 3D rendering images captured at the cell adhesion surface (Figure 3A). The count of cell spreading on the PDMS dishes revealed that mock-transfected 143B cells preferentially spread over the lung-mimicking 1.5 kPa substrate and that Lamin A/C over-expression changes this preference in favor to the 28 kPa substrate (Figure 3B). A further assessment of cell spreading by cell area measurement demonstrated that mock-transfected 143B are statistically larger, thereby more spread, on the 1.5 kPa substrate than LMNA-transfected cells, while the opposite condition is observed on the 28 kPa substrate (Figure 3C). To assess if other factors are required in the modulation of the mechanical signal in LMNA-transfected cells, the Integrin β-3 subunit (ITGB3) and the α-tubulin (α-TUB) were evaluated as an index of cell membrane and cytoskeleton involvement, respectively. Figure 3D shows that in those conditions in which cells resulted more adherent to the substrate (LMNA-transfected cells on 28 kPa substrate and Mock-transfected cells on 1.5 kPa substrate), the levels of ITGB3 and α-TUB were higher than the counterparts, thereby suggesting their involvement in the cell adhesion mediated by LMNA gene expression. Moreover, the analysis of percentage of cell with filopodia and the filopodia content as parameters of cell attachment on a surface confirmed the preferential adhesion and spreading of mock-transfected 143B cells on 1.5 kPa PDMS substrate while 143B cells became strongly adherent to the 28 kPa PDMS substrate following Lamin A/C over-expression (Figure 4). All together, these results confirmed an overall modulation of 143B adhesion properties following Lamin A/C reintegration.

## Discussion

Lamin A/C as mechanosensors are arousing growing interest in the field of mechanopathology, given their well-recognized involvement in the basic processes of cell biology and their altered expression in several pathological conditions, other than the obvious laminopathies (Chen et al., 2009; Mewborn et al., 2010; Puckelwartz et al., 2011; Dantas et al., 2021). In particular, Lamin A/C deregulation has been related to cancer onset and progression (Irianto et al., 2016; Dubik and Mai, 2020), but with conflicting results in dependance on the specific tumor assessed (Foster et al., 2010). Indeed, reduced expression of Lamin A/C assessed by immunohistochemistry on tissue slides has been associated to poor prognosis in both gastric cancer (Wu et al., 2009) and early stage breast cancer (Alhudiri et al., 2019) patients. On the contrary, Lamin A/C levels are described as risk biomarkers for colorectal cancer patients, given that patients with A-type lamin-expressing tumors have significantly worse prognosis than patients with A-type lamin negative tumors and Lamin A/C overexpression in a colorectal cancer cell line increases cell motility and invasion by down-regulating cell adhesion (Willis et al., 2008). In parallel, the work by Kong et al. (2012) clearly demonstrated that Lamin A/C is positively involved in malignant behavior of prostate cancer cells, showing increased Lamin A/C proteins expression in high-risk cancer patients and that RNA knockdown or overexpression of Lamin A/C in prostate cancer cell lines resulted in inhibition or stimulation, respectively, of cell growth, colony formation, migration and invasion. Taken together, these works clearly demonstrate that Lamin A/C cannot be uniquely defined as cancer stimulating or counteracting factors. In our opinion, these conflicting findings depend on the basal Lamin A/C expression, very different among the several tissues. As described by Swift et al. (2013), any tissue have a specific elasticity that correlates with the basal Lamin A/C expression and with the Lamin A/C:Lamin B ratio. In view of this, the oncogenic transformation could induce a deregulated expression of Lamin A/C in tumor cells and any alteration from its basal condition, intended as either stimulating or inhibitory, could greatly influence the homeostasis of the tumor microenvironment. In support of this, our previous work demonstrates that within osteosarcoma specimens Lamin A/C varies greatly and in dependance on cancer cell aggressiveness (Urciuoli et al., 2020). In support of our work, Evangelisti et al. (2020) have confirmed that the condition of low Lamin A expression confers to osteosarcoma cells a significant increase in migration potential, while overexpression of Lamin A reduces their migration ability. Here, we deepened Lamin A/C involvement in osteosarcoma cell tumoral behavior, showing that its reintegration in high metastatic 143B cells, expressing basal low levels of Lamin A/C, restores cell adhesion properties as well as tumor features of low aggressive non-metastatic osteosarcoma cells. Specifically, in this work we used PDMS substrates resembling the elasticity of bone osteoid and lung parenchyma matrices (i.e., 28 and 1.5 kPa, respectively) in order to demonstrate that cancer cell tropism depends on Lamin A/C expression. Indeed, mock-transfected 143B cells show a preferential adhesion and spreading on lung-resembling 1.5 kPa PDMS substrate, as assessed by phalloidin, ITGB3 and α-TUB staining and filopodia and cell area analyses. As regarding phalloidin staining, based on the distribution along the cell membrane and according to the literature (Jacquemet et al., 2015), we can speculate the actin filaments are filopodia. Indeed, in 2D culture condition invadopodia are usually present beneath the cell body, since they are involved in the degradation and the invasion of the matrix. In our condition, actin dots are present all around the cells, so we can speculate they are filopodia involved in cell anchorage on the substrate. The behavior of 143B cells is completely reversed after Lamin A/C reintegration, as LMNA- transfected 143B cells can still adhere on 1.5 kPa PDMS substrate but 24 h after culturing most of them resulted round shaped and with a low filopodia content, and reduced amount of ITGB3 and α-TUB. It worth noting that Lamin A/C reintegration is also responsible in reducing 143B cell aggressiveness, as demonstrated by decreased proliferation rate and migration capability.

In conclusion, our work demonstrates that modulating Lamin A/C expression is responsible in influencing osteosarcoma cell aggressiveness and adhesion properties on specific matrices. These findings, in our opinion, could represent a starting point for future investigations that will have to consider the mechano-environment of osteosarcoma as a crucial factor in the onset, development and progression of this bone tumor. Although further studies on different cell types are needed to confirm our hypothesis, this work provides a first evidence about the relationship between intrinsic Lamin A/C expression and a specific tumor cell tropism.

## Data Availability Statement

The original contributions presented in the study are included in the article/supplementary material, further inquiries can be directed to the corresponding author/s.

## Author Contributions

EU and VD’O: data generation. EU, SP, and BP: data analysis. BP: supervision and funding acquisition. EU and BP: original draft preparation for the manuscript. All authors contributed to manuscript revision, read, and approved the submitted version.

## Conflict of Interest

The authors declare that the research was conducted in the absence of any commercial or financial relationships that could be construed as a potential conflict of interest.

## Publisher’s Note

All claims expressed in this article are solely those of the authors and do not necessarily represent those of their affiliated organizations, or those of the publisher, the editors and the reviewers. Any product that may be evaluated in this article, or claim that may be made by its manufacturer, is not guaranteed or endorsed by the publisher.

## Footnotes

1
http://n2t.net/addgene:17662
